# Video gaming addiction and its association with memory, attention and learning skills in Lebanese children

**DOI:** 10.1186/s13034-020-00353-3

**Published:** 2020-12-12

**Authors:** Youssef Farchakh, Chadia Haddad, Hala Sacre, Sahar Obeid, Pascale Salameh, Souheil Hallit

**Affiliations:** 1 Faculty of Medicine and Medical Sciences, Holy Spirit University of Kaslik (USEK), Jounieh, Lebanon; 2 Research and Psychology Departments, Psychiatric Hospital of the Cross, P.O. Box 60096, Jal Eddib, Lebanon; 3 INSERM, Univ. Limoges, CH Esquirol, IRD, U1094 Tropical Neuroepidemiology, Institute of Epidemiology and Tropical Neurology, GEIST, Limoges, France; 4 INSPECT-LB: Institut National de Santé Publique, Epidémiologie Clinique Et Toxicologie-Liban, Beirut, Lebanon; 5 Faculty of Arts and Sciences, Holy Spirit University of Kaslik (USEK), Jounieh, Lebanon; 6 Faculty of Pharmacy, Lebanese University, Beirut, Lebanon; 7 Faculty of Medicine, University of Nicosia, Nicosia, Cyprus

**Keywords:** Video gaming, Addiction, Learning, Attention, Memory

## Abstract

**Background:**

Examining whether any association exists between addiction to video games and cognitive abilities in children could inform ongoing prevention and management of any possible harm. The objective of this study was to investigate the associations between addiction to video games, and memory, attention and learning abilities among a sample of Lebanese school children.

**Methods:**

This cross-sectional study, conducted between January and May 2019, enrolled 566 school children aged between 9 and 13 years. Three private schools were chosen conveniently for this study. Students were randomly chosen from the list given by the school administration. The students’ parents are those who responded to the questionnaire.

**Results:**

The results showed that higher addiction to video gaming salience was significantly associated with worse episodic memory, problem solving, basic reading skills, written expression skills and worse clinical attention. Higher addiction to video gaming tolerance were significantly associated with worse novel problem solving and worse attention. Higher addiction to video gaming withdrawal were significantly associated with worse attention, factual memory, attention, processing speed, visual spatial organization, sustained sequential processing, working memory, novel problem solving and worse written expression skills.

**Conclusion:**

The results suggest a correlation between addiction to video games and worse memory, attention, as well as cognitive and academic abilities among school children. Those findings indicate the need for more extensive research, and serve to highlight vital next steps needed in future papers, such as identifying predicting factors that could aid in early detection of video gaming addiction in children.

## Background

Recent advances in new technologies have made video games the top leisure time occupation for children, who are particularly susceptible for addiction. Currently, video games have become the most famous type played worldwide among children. Nielsen reports that total weekly time spent playing games increased expeditiously from 5.1 to 6.3 h in 2011 and 2013 respectively [1]. In a study involving children aged between 9 and 12, from 12 different countries, it was found that 8.6 h per day were spent on playing video games [2]. Importantly, the emerging phenomenon of video game addiction represents a real and potential widespread problem that defies easy solutions and prevention methods, and requires further investigations, especially in the childhood population.

In fact, despite the benefits that video games have, such as socialization and entertainment, empirical and clinical studies have frequently demonstrated that the excessive use of video games may drive to adverse consequences in miscellaneous areas of psychological development and can result in an addiction among a small portion of gamers [3]. Impaired control over gaming and increasing priority over daily activities and other life interests could manifest as an evidence of gaming addiction [4]. In its fifth edition, the Diagnostic and Statistical Manual of Mental Disorders (DSM-5) identified video game addiction in the form of internet gaming disorder, as a condition in need of supplementary studies [5]. Additionally, the 11th revision of International Classification of Diseases (ICD-11) defined gaming disorder as a recurrent gaming behavior pattern that carries both offline and online gaming [6]. Science suggests that addictions arise from a mixture of a genetic predisposition and a repeated exposure to a particular substrate [7].

Along with that, there has been growing public concerns about potential adverse effects, including the possibility that video games might affect memory in children [8]: as an important process for comprehension, memory updating and working memory, among other cognitive skills, have usually been reported to be damaged across different behavioral disorder individuals and addictive populations [9]. Even though there are some studies that investigate the effect of video games on cognitive functions and academic performance in children [10, 11], consequences on memory are still a prevalent subject of debate.

Although video gaming is a free time activity, it does cause problems for some children, affecting their attention competencies. Both a meta-analysis and a systematic review conducted by Ho et al. and Carli et al. respectively suggested an association between inattention and internet/gaming addiction [12]. Moreover, plenty of other studies supported this finding and proved a strong correlation between the severity of inattention in attention deficit hyperactivity disorder (ADHD) and internet/gaming addiction [13], even after regulating the effects of depression and anxiety symptoms, as well as personality traits [12].

Unsurprisingly, video gaming advancements have led to concerns from parents and educators that the exaggerated amount of time consumed by children on video games, may result in decreased learning and academic abilities [11]. Handful of studies have generated inconclusive results regarding a possible association between poor school grades and problematic gaming [11, 14]. Furthermore, in cross-sectional studies, pathological video gaming has been connected to a range of negative consequences, such as lower school and academic performances, but the need for further research to explore this finding is mandatory [11]. Moreover, researchers have been attempting to shed light on addiction to video games and its association with children’s development and behaviors. Despite all the efforts made, the literature still lacks sufficient studies on the subject.

In Lebanon, adolescents showed a 43.6% of moderate to severe problematic internet use [15]. Children are affected by video gaming and its related addiction. According to a Lebanese research study published in 2018, gaming disorder was associated with being younger. Moreover, Lebanese children are witnessing devastating long-term adverse effects on their behavioral, physical and psychological health, due to the rapidly advancing technology, which makes it very essential to focus on this particular Lebanese age group [14]. From the research point of view, only three Lebanese studies were detected concerning video games addiction [14, 16, 17]. Two of them worked on the development and validation of specific scales for video games addiction and internet gaming disorder [15, 16], while the third one explored the relationships between gaming disorders, sleep habits, and academic achievement in Lebanese adolescents [14]. The last study showed that the pooled prevalence of internet gaming disorder was 9.2% [14]. Examining whether any association exists between addiction to video games and cognitive abilities in children could inform ongoing prevention and management of any possible harm. The objective of this study was to investigate the associations between addiction to video games, and memory, attention and learning abilities among a sample of Lebanese schoolchildren.

## Methods

### Participants

This study was a cross-sectional, conducted between January and May 2019. Three private schools were chosen conveniently for this study. Students were randomly chosen from the list given by the school administration. All participants between the age of 9 and 13 years of age were eligible to participate. The students’ parents are those who answered the questionnaire. Prior to participation, parents were briefed on the study objectives and methodology, and were assured of the anonymity of their participation. Parents had the right to accept or refuse participation in the study, with no financial compensation provided in return.

### Sample size calculation

The Epi info program [Centers for Disease Control and Prevention (CDC), Epi Info™] was employed for the calculation of the required sample size for our study, with a prevalence of internet gaming disorder of 9.2% among 524 Lebanese high school students from a study done by Hawi et al. [14], with an acceptable margin of error of 5% and design effect of 2. The youth population is estimated to be 585,000 according to the UNDP statistics in Lebanon; the result showed that the biggest required sample size is 256 participants.

### Procedure

The questionnaire was distributed to each student in the classroom to be taken home. Parents filled it within 25 min approximately. The completed questionnaires were collected back and sent for data entry. During the data collection process, the anonymity of the participants was guaranteed.

### Questionnaire

The self-administered questionnaire used was in Arabic, the native language of Lebanon. The first part assessed the sociodemographic details of the participants (i.e. age, gender, grade, and father and mother education level). The second part of the questionnaire included the following scales:

### Game Addiction Scale for Children (GASC)

The 21-item Gaming Addiction Scale (GASC) is an instrument based on DSM criteria to assess gaming addiction. The seven items in the GAS are rated using a five-point Likert scale ranging from 1 (never) to 5 (very often). A higher score on the GAS indicates more problematic use of online gaming. The scale measures 7 criteria of computer addiction: salience, tolerance, mood modification, withdrawal, relapse, conflict and problems [18]. In this study, the Cronbach alpha values for the GAS was 0.948.

### Children’s Memory Questionnaire (CMQ)

The CMQ is a 36-item questionnaire designed to assess parents’ perceptions of their children’s memory. The CMQ requires parents to assess their child’s memory based on five possible options: 1 = never or almost never happens; 2 = happens less than once a week; 3 = happens once or twice in a week; 4 = happens about once a day; and 5 = happens more than once a day [19]. Three subscales derived from the total scale representing the episodic memory, visual memory and working memory and attention. The higher the scores, the greater the impairment in the cognition domain [19]. In this study, the Cronbach alpha values for the episodic memory subscale was 0.888, for the visual memory was 0.770 and for the working memory was 0.845.

### Clinical attention problems scale

The scale measures the frequency of activity and attention by asking the parent and teacher to respond to a series of 12 statements and their applicability to their child in the morning and afternoon. Response options range from “not true” (0), “somewhat or sometimes true” (1), “very often” or often true (2). The higher the scores, the greater the attention problems exist [20]. In this study, the Cronbach alpha values for the clinical attention problem in the morning and in the afternoon were 0.844 and 0.839 respectively.

### Learning, Executive and Attention Functioning (LEAF) Scale

The LEAF is a 55 item self-report questionnaire that assesses executive functions, related neurocognitive functions, and academic skills in children and adults. The LEAF evaluates a broad set of core cognitive abilities as well as related cognitive learning and academic abilities. Cognitive areas assessed by the LEAF include attention, processing speed (including visual-spatial organization skills), and sustained sequential processing to achieve goals (e.g., planning and executing goal-directed behavior), working memory, and novel problem-solving. Also, LEAF includes comprehension and concept formation, declarative/factual memory, and academic functioning. The LEAF contains Academic subscales assessing reading, writing, and math fluency and abilities. LEAF items are grouped by subscale, and all subscales have the same number of items.

The subscales of the LEAF are: (1) comprehension and conceptual learning (tracking and understanding information), (2) factual memory (memorization and retention of facts); (3) attention (sustained focus); (4) processing speed (speed of completing cognitive and behavioral tasks that involve a component of focus and concentration); (5) visual-spatial organization (organization and visual-constructive skills); (6) sustained sequential processing (planning and sustaining effort in order to follow and complete multistep directions and sequences); (7) working memory (remembering and processing multiple things at the same time); and (8) novel problem solving (initiating effort toward processing new or unfamiliar information). (9) Mathematics skills (math calculation difficulty); (10) basic reading skills (reading/phonics difficulty); and (11) written expression skills (limited/impoverished or slow/effortful written expression). Individual items are rated on a 0–3 scale, and a raw subscale score for each of the 11 content areas is created by summing the 5 constituent items, such that higher scores indicate more cognitive problems [21]. In this study, the Cronbach alpha values for the subscales was: comprehension and conceptual learning = 0.961; factual memory = 0.792; attention = 0.901; processing speed = 0.866; visual-spatial organization = 0.729; sustained sequential processing = 0.768; working memory = 0.816; novel problem solving = 0.811; mathematics skills = 0.871; basic reading skills = 0.923 and written expression skills = 0.905.

### Translation procedure

The forward translation was done by one translator. An expert committee formed by healthcare professionals and a language professional verified the Arabic translated version. A backward translation was then performed by a second translator, unaware of the initial English version. The back-translated English questionnaire was subsequently compared to the original English one, by the expert committee. Discrepancies related to inadequate expressions and concepts, confusing in meaning and slightly off in meaning during the reconciliation of the back translated questionnaire with the original source were resolved by consensus.

### Statistical analysis

SPSS software version 23 was used to conduct data analysis. Cronbach’s alpha values were recorded for reliability analysis for all the scales. A descriptive analysis was done using the counts and percentages for categorical variables and mean and standard deviation for continuous measures. A multivariate analysis of covariance (MANCOVA) was carried out to compare multiple measures (each scale was taken as a dependent variable) taking the GAS as the major independent variable, controlling for potential confounding variables: age, gender, family monthly income, and mother and father education level. A p-value less than 0.05 was considered significant.

## Results

Out of 700 distributed questionnaires, 566 (80.86%) questionnaires were completed and collected back. The sociodemographic characteristics of the participants are summarized in Table 1. The mean age was 10.77 ± 1.38 years, with 55.2% male. Also, higher education level in parents was found in 58.9% among mother and 39.4% among father.

**Table 1:** Sociodemographic characteristics of the study sample

	Frequency	Percentage (%)
Gender		
Male	286	55.2
Female	232	44.8
Education level father		
Illiterate	14	2.9
Primary	34	7.0
Complementary	111	22.9
Secondary	135	27.8
University	191	39.4
Education level mother		
Illiterate	14	2.9
Primary	11	2.2
Complementary	58	11.9
Secondary	118	24.1
University	288	58.9

	Mean	SD
Age (years)	10.77	1.38

### Description of the scales used

The description of all the scales used in terms of mean, standard deviation, median, minimum and maximum is summarized in Table 2.

**Table 2:** Description of the scales used

Scale	Mean	Standard deviation	Median	Minimum	Maximum
Episodic memory	6.32	7.06	4	0	46
Working memory	3.99	5.45	2	0	32
Visual memory	2.38	3.86	1	0	27
Clinical attention morning	16.75	4.56	16	0	34
Clinical attention afternoon	17.22	4.96	16	0	36
Comprehension conceptual learning	2.14	2.78	1	0	15
Factual memory	1.98	2.51	1	0	15
Attention	2.56	3.34	1	0	15
Processing speed	2.75	3.30	2	0	15
Visual spatial organization	2.75	2.85	2	0	16
Sustained sequential processing	2.74	2.81	2	0	14
LEAF working memory	2.81	2.84	2	0	15
Novel problem solving	2.50	2.72	2	0	15
Mathematical skills	2.19	2.97	1	0	15
Basic reading skills	2.64	3.30	1	0	15
Written expression skills	3.53	3.55	3	0	15
Addiction video gaming salience	3.95	3.56	3	0	12
Addiction video gaming tolerance	3.85	3.56	3	0	12
Addiction video gaming mood modification	5.00	3.95	4	0	12
Addiction video gaming relapse	5.56	3.75	6	0	12
Addiction video gaming withdrawal	4.16	3.29	4	0	12
Addiction video gaming conflict	2.85	2.88	2	0	12
Addiction video gaming problems	1.39	2.22	0	0	12

### Bivariate analysis

The bivariate analysis of the sociodemographic variables associated with the memory and attention scores is summarized in Tables 3 and 4.

**Table 3:** Bivariate analysis of factors associated with the memory and attention scores

Variable	Episodic memory	Working memory	Visual memory	Clinical attention morning	Clinical attention afternoon	Comprehension conceptual learning	Factual memory	Attention	Processing speed
Gender									
Male	6.65 ± 7.03	4.42 ± 5.80	2.36 ± 3.68	17.14 ± 4.65	17.72 ± 5.13	2.26 ± 3.01	2.04 ± 2.60	2.82 ± 3.60	2.89 ± 3.47
Female	5.84 ± 7.13	3.38 ± 4.90	2.33 ± 3.97	16.21 ± 4.40	16.55 ± 4.64	2.00 ± 2.50	1.85 ± 2.34	2.19 ± 2.95	2.52 ± 3.05
*p*	0.209	*0.034*	0.932	*0.028*	*0.011*	0.307	0.405	*0.041*	0.224
Educational level									
Illiterate/primary	7.87 ± 9.52	6.15 ± 7.80	2.83 ± 3.59	17.70 ± 5.39	18.28 ± 5.53	3.32 ± 3.37	3.09 ± 3.15	4.15 ± 4.38	3.61 ± 3.75
Complementary	6.22 ± 7.24	4.81 ± 5.64	2.31 ± 3.49	17.19 ± 4.35	17.72 ± 5.08	2.56 ± 2.87	2.08 ± 2.58	3.16 ± 3.63	3.41 ± 3.68
Secondary	5.52 ± 6.43	3.53 ± 4.64	2.14 ± 3.79	16.98 ± 4.74	17.25 ± 4.74	2.06 ± 2.59	2.00 ± 2.36	2.52 ± 3.32	2.95 ± 3.39
University	6.28 ± 6.53	2.97 ± 4.58	2.10 ± 3.52	16.02 ± 4.23	16.66 ± 4.72	1.45 ± 2.40	1.52 ± 2.28	1.73 ± 2.56	1.95 ± 2.68
*p*	0.273	*0.001*	0.645	0.054	0.146	*< 0.001*	*0.002*	*< 0.001*	*< 0.001*
Mother’s educational level									
Illiterate/primary	4.50 ± 4.05	6.08 ± 6.85	3.58 ± 4.48	16.65 ± 5.73	17.83 ± 5.16	3.78 ± 4.38	3.09 ± 3.28	3.00 ± 3.90	4.09 ± 4.57
Complementary	6.65 ± 7.67	5.28 ± 6.17	2.89 ± 3.32	17.89 ± 4.65	18.52 ± 5.49	3.07 ± 3.27	2.94 ± 3.20	4.07 ± 4.00	3.93 ± 3.35
Secondary	6.39 ± 8.47	3.92 ± 5.66	2.09 ± 3.98	16.75 ± 4.90	17.23 ± 5.24	2.06 ± 2.61	1.62 ± 1.88	2.37 ± 3.05	2.97 ± 3.38
University	6.31 ± 6.53	3.47 ± 4.88	2.13 ± 3.67	16.53 ± 4.26	16.96 ± 4.61	1.74 ± 2.40	1.77 ± 2.43	2.29 ± 3.21	2.32 ± 3.07
*p*	0.639	*0.025*	0.162	0.262	0.187	*< 0.001*	*0.001*	*0.003*	*0.001*

Italic values indicate significant *p*-values

**Table 4:** Bivariate analysis of factors associated with the memory and attention scores

Variable	Visual spatial organization	Sustained sequential processing	LEAF working memory	Novel problem	Mathematics skills	Basic reading skills	Written expression skills
Gender							
Male	2.96 ± 3.00	2.94 ± 2.93	2.94 ± 3.09	2.57 ± 2.88	1.97 ± 2.78	2.89 ± 3.56	3.76 ± 3.82
Female	2.44 ± 2.60	2.45 ± 2.62	2.60 ± 2.49	2.36 ± 2.48	2.45 ± 3.16	2.24 ± 2.80	3.21 ± 3.19
*p*	*0.046*	0.059	0.194	0.413	0.086	*0.026*	0.09
Father’s educational level							
Illiterate/primary	3.54 ± 3.54	3.60 ± 3.44	3.83 ± 3.73	3.08 ± 3.23	3.44 ± 3.91	4.02 ± 4.15	4.70 ± 4.11
Complementary	3.25 ± 3.26	3.08 ± 3.07	3.21 ± 3.00	2.96 ± 2.95	2.42 ± 3.10	3.03 ± 3.21	4.25 ± 3.79
Secondary	2.62 ± 2.64	2.82 ± 2.93	2.74 ± 2.80	2.63 ± 2.74	2.22 ± 2.91	2.60 ± 3.29	3.68 ± 3.83
University	2.26 ± 2.34	2.19 ± 2.24	2.21 ± 2.31	1.92 ± 2.40	1.53 ± 2.32	1.92 ± 2.78	2.64 ± 2.80
*p*	*0.006*	*0.006*	*0.001*	*0.005*	*< 0.001*	*< 0.001*	*< 0.001*
Mother’s educational level							
Illiterate/primary	3.59 ± 3.81	3.13 ± 2.38	3.41 ± 2.97	2.82 ± 2.50	2.73 ± 2.86	3.32 ± 2.61	3.64 ± 3.50
Complementary	3.46 ± 3.21	3.77 ± 3.58	3.71 ± 3.62	3.70 ± 3.47	3.28 ± 3.33	2.91 ± 2.84	4.96 ± 4.09
Secondary	2.96 ± 3.01	3.04 ± 2.94	2.57 ± 2.58	2.27 ± 2.44	2.19 ± 3.32	2.81 ± 3.39	3.52 ± 3.63
University	2.45 ± 2.53	2.41 ± 2.58	2.62 ± 2.72	2.31 ± 2.68	1.85 ± 2.63	2.41 ± 3.32	3.23 ± 3.37
*p*	*0.026*	*0.004*	*0.035*	*0.005*	*0.007*	0.404	*0.012*

Post hoc analysis: Father’s education level: working memory (illiterate/primary vs secondary p = 0.021; illiterate/primary vs university p = 0.002; complementary vs university p = 0.026); comprehension conceptual morning (illiterate/primary vs secondary p = 0.036; illiterate/primary vs university p < 0.001; complementary vs university p = 0.005); Factual memory (illiterate/primary vs university p = 0.001); attention (illiterate/primary vs secondary p = 0.022; illiterate/primary vs university p < 0.001; complementary vs university p = 0.003); processing speed (illiterate/primary vs university p = 0.014; complementary vs university p = 0.002); visual spatial organization (illiterate/primary vs university p = 0.034; complementary vs university p = 0.026); sustained sequential processing (illiterate/primary vs university p = 0.014); working memory (illiterate/primary vs university p = 0.003; complementary vs university p = 0.024); novel problem solving (complementary vs university p = 0.013); mathematics skills (illiterate/primary vs university p < 0.001); basic reading skills (illiterate/primary vs university p < 0.001; complementary vs university p = 0.033); written expression (illiterate/primary vs university p = 0.002; complementary vs university p = 0.001). Mother’s education level: comprehension conceptual learning (illiterate/primary vs secondary p = 0.033; illiterate/primary vs university p = 0.003; complementary vs university p = 0.005); Factual memory (complementary vs secondary p = 0.006; complementary vs secondary p = 0.008); LEAF attention (complementary vs secondary p = 0.012; complementary vs university p = 0.002); processing speed (complementary vs university p = 0.005); sustained sequential processing (complementary vs university p = 0.005); novel problem solving (complementary vs secondary p = 0.009; complementary vs secondary p = 0.004); mathematics skills (complementary vs university p = 0.005); written expression (complementary vs university p = 0.006)

Italic values indicate significant *p*-values

Higher addiction to video gaming salience, tolerance, relapse, withdrawal, conflict and problems scores were significantly correlated with lower/worse episodic memory, working memory, visual memory, clinical attention in the morning and in the afternoon, comprehension and conceptual learning, factual memory, LEAF attention, processing speed, visual spatial organization, sustained sequential processing, LEAF working memory, novel problem solving, mathematics skills, basic reading and written expression skills scores, except for the mood modification that was not associated with the visual memory and mathematics skills scores (Table 5).

**Table 5:** Bivariate analysis of continuous variables associated^d^ with the video gaming addiction subscales scores

Variable	Addiction video gaming salience	Addiction video gaming tolerance	Addiction video gaming mood modification	Addiction video gaming relapse	Addiction video gaming withdrawal	Addiction video gaming conflict	Addiction video gaming problems
Episodic memory	0.363^c^	0.353^c^	0.216^c^	0.263^c^	0.293^c^	0.325^c^	0.244^c^
Working memory	0.250^c^	0.253^c^	0.169^c^	0.211^c^	0.298^c^	0.299^c^	0.312^c^
Visual memory	0.137^b^	0.133^b^	0.069	0.119^b^	0.209^c^	0.274^c^	0.343^c^
Clinical attention morning	0.361^c^	0.305^c^	0.278^c^	0.320^c^	0.343^c^	0.329^c^	0.232^c^
Clinical attention afternoon	0.334^c^	0.283^c^	0.248^c^	0.300^c^	0.323^c^	0.300^c^	0.231^c^
Comprehension conceptual learning	0.201^c^	0.210^c^	0.154^b^	0.143^b^	0.242^c^	0.200^c^	0.226^c^
Factual memory	0.204^c^	0.241^c^	0.138^b^	0.179^c^	0.296^c^	0.291^c^	0.258^c^
LEAF attention	0.287^c^	0.277^c^	0.211^c^	0.228^c^	0.293^c^	0.246^c^	0.218^c^
Processing speed	0.264^c^	0.253^c^	0.169^c^	0.210^c^	0.302^c^	0.245^c^	0.215^c^
Visual spatial organization	0.337^c^	0.312^c^	0.210^c^	0.255^c^	0.338^c^	0.287^c^	0.173^c^
Sustained sequential processing	0.282^c^	0.284^c^	0.222^c^	0.282^c^	0.329^c^	0.222^c^	0.152^b^
LEAF working memory	0.263^c^	0.243^c^	0.198^c^	0.213^c^	0.295^c^	0.235^c^	0.177^c^
Novel solving problem	0.226^c^	0.185^c^	0.175^c^	0.164^c^	0.272^c^	0.218^c^	0.155^b^
Mathematics skills	0.148^b^	0.133^b^	0.081	0.099^a^	0.140^b^	0.130^b^	0.160^b^
Basic reading skills	0.270^c^	0.230^c^	0.150^b^	0.202^c^	0.232^c^	0.209^c^	0.160^b^
Written expression	0.310^c^	0.289^c^	0.191^c^	0.214^c^	0.293^c^	0.217^c^	0.107^a^

^a^*p* < 0.05; ^b^*p* < 0.01; ^c^*p* < 0.001

### Multivariate analysis

The MANCOVA analysis was performed taking the scales as the dependent variables and the addiction to video gaming salience as the independent variable, adjusting for the covariates (age, gender, family monthly income, and mother and father education levels).

Higher addiction to video gaming salience was significantly associated with higher episodic memory score (worse episodic memory), higher novel problem solving score (worse problem solving), higher basic reading skills score (worse basic reading skills), higher written expression skills score (worse written expression skills) and higher clinical attention in the morning and afternoon scores (worse episodic memory and attention).

Higher addiction to video gaming tolerance were significantly associated with higher novel problem solving score (worse novel problem solving) and higher clinical attention in the morning and afternoon scores (worse attention).

Higher addiction to video gaming withdrawal were significantly associated with higher clinical attention in the afternoon score (worse attention), higher factual memory score (worse factual memory), higher attention score (worse attention), higher processing speed score (worse processing speed), higher visual spatial organization score (worse visual spatial organization), higher sustained sequential processing score (worse sustained sequential processing), higher working memory score (worse working memory), higher novel problem solving score (worse novel problem solving) and higher written expression skills score (worse written expression skills) (Table 6).

**Table 6:** Multivariate analysis of covariance (MANCOVA)

	Beta	Partial Eta squared	p-value	95% confidence interval	
				Lower bound	Upper bound
Episodic memory					
Addiction video gaming salience	0.581	0.024	0.009	0.149	1.013
Working memory					
Addiction video gaming salience	0.292	0.013	0.058	− 0.010	0.594
Addiction video gaming mood modification	0.213	0.012	0.061	− 0.010	0.437
Father university compared to illiteracy/primary level*	− 1.886	0.012	0.070	− 3.929	0.157
Mother university compared to illiteracy/primary level*	− 2.522	0.011	0.077	− 5.315	0.272
Visual memory					
Mother secondary compared to illiteracy/primary level*	− 2.204	0.024	0.009	− 3.851	− 0.558
Addiction video gaming withdrawal	0.139	0.010	0.088	− 0.021	0.299
Clinical attention morning					
Addiction video gaming salience	0.562	0.057	< 0.001	0.294	0.830
Addiction video gaming tolerance	0.411	0.026	0.007	0.114	0.708
Father university compared to illiteracy/primary level*	− 2.052	0.017	0.027	− 3.866	− 0.238
Clinical attention afternoon					
Addiction video gaming salience	0.561	0.049	0.001	0.270	0.851
Addiction video gaming tolerance	0.367	0.018	0.025	0.046	0.689
Addiction video gaming withdrawal	0.293	0.016	0.035	0.020	0.565
LEAF comprehension conceptual learning					
Father university compared to illiteracy/primary level*	− 1.445	0.021	0.006	− 2.464	− 0.425
Mother university compared to illiteracy/primary level*	− 1.348	0.017	0.055	− 2.723	0.028
LEAF factual memory					
Addiction video gaming withdrawal	0.156	0.019	0.019	0.026	0.286
Father university compared to illiteracy/primary level*	− 1.103	0.011	0.015	− 1.988	− 0.218
Mother secondary compared to illiteracy/primary level*	− 1.724	0.027	0.006	− 2.960	− 0.489
Mother university compared to illiteracy/primary level*	− 1.357	0.026	0.026	− 2.551	− 0.164
LEAF attention					
Addiction video gaming withdrawal	0.172	0.012	0.043	0.005	0.339
Father university compared to illiteracy/primary level*	− 1.660	0.021	0.014	− 2.988	− 0.332
LEAF processing speed					
Addiction video gaming withdrawal	0.245	0.023	0.011	0.057	0.434
LEAF visual spatial organization					
Addiction video gaming withdrawal	0.145	0.013	0.044	0.004	0.285
LEAF sustained sequential processing					
Addiction video gaming withdrawal	0.244	0.032	0.001	0.098	0.389
LEAF working memory					
Addiction video gaming withdrawal	0.183	0.022	0.014	0.037	0.329
Father university	− 1.254	0.006	0.019	− 2.302	− 0.205
LEAF novel problem solving					
Addiction video gaming salience	0.212	0.025	0.008	0.056	0.369
Addiction video gaming tolerance	0.214	0.021	0.016	0.041	0.388
Addiction video gaming withdrawal	0.233	0.033	0.002	0.086	0.379
LEAF mathematic skills					
Gender (females vs males*)	0.849	0.020	0.018	0.148	1.551
Father secondary level of education compared to illiteracy/primary level*	− 1.319	0.015	0.040	− 2.578	− 0.059
Father university level of education compared to illiteracy/primary level*	− 1.534	0.021	0.015	− 2.769	− 0.299
LEAF basic reading skills					
Addiction video gaming salience	0.220	0.016	0.035	0.015	0.425
Father university level of education compared to illiteracy/primary level*	− 1.919	0.026	0.007	− 3.305	− 0.533
LEAF written expression skills					
Addiction video gaming salience	0.236	0.012	0.019	0.038	0.433
Addiction video gaming withdrawal	0.231	0.016	0.015	0.044	0.417
Father university	− 1.917	0.022	0.005	− 3.256	− 0.577

*Reference group

## Discussion

Thus far, this is the first study to be conducted on Lebanese school children to evaluate the association between addiction to video games, memory, attention and learning abilities. Taking into consideration that intensive video gaming has frequently been linked to the development of addictive-like behaviors among children, we found highly important to assess some cognitive factors that could be affected in this delicate population. The results showed that higher addiction to video gaming was significantly associated with higher memory score (worse memory), with higher attention score (worse attention), and higher LEAF scale and subscales scores (worse cognitive and academic abilities). We also found that having a father with university education level was significantly associated with lower attention score (better attention) and lower LEAF scale and subscales scores (better cognitive and academic abilities).

Higher addiction to video gaming was significantly associated with higher memory score (worse memory). When the different studies were reviewed, the results were found to be contradictory. Some of the studies argued that video games have negative effects on memory, while other ones did not support this finding [22]. Moreover, an observational study comparing patients with internet gaming disorder against healthy control groups revealed that the formers had lower working memory functioning [8]. This particular result regarding memory may relate to the fact that spending too much time playing the same type of games could be harming their abilities to retain and boost memories.

Moreover, higher addiction to video gaming was significantly associated with higher attention score (worse attention). Similar to our results, increasing behavioral studies proved that video games addicts showed attention deficits. De facto, Zhou et al. [23] demonstrated that when performing the attention-related task, more total error rates was observed in subjects with internet gaming addiction in comparison to controls. To boot, Song et al. [24] established a positive correlation between inattention and internet gaming addiction severity, while lack of attention was verified to be a high predictor of online gaming addiction by Ko et al. [25]. The finding we obtained could be interpreted by the instant rewards and constant stimulation of video games that raise the threshold for children to pay attention in less stimulating situations, where working harder is required to get rewards. Another theory could be that children suffering from attentional problems become captivated by video games as a coping strategy of their behavioral disorder.

Likewise, higher addiction to video gaming was significantly associated with higher LEAF scale and subscales scores (worse cognitive and academic abilities). Correspondingly, Gentile et al. [26] found that video games addiction among Singaporean adolescents predicted worse school grades and cognitive well-being. In contrast, another study conducted by Regina et al. [11] demonstrated no evidence that excessive video gaming negatively affects adolescents’ school performances. Our result may be understood in the light of the fact that more time spent on video games means less time spent on academic activities, as well as less hours of sleep overall, which generally make children with heavy video gaming attitudes, less alert and more susceptible to cognitive errors.

In addition to the previous findings, having a father with university education level was significantly associated with lower attention score (better attention) and lower LEAF scale and subscales scores (better cognitive and academic abilities). To our knowledge, no study in the literature discussed the education level of the parents, particularly the father, in this particular context. The cause for our result could be that highly educated fathers raise their children in a similar educative way, which enhances their cognitive and academic skills.

### Clinical implications

The current study provides many valuable contributions to the literature. Despite growing numbers of published studies examining cognitive skills in children playing video games, there is a paucity of rigorous ones from which to draw firm conclusions. This study procures firm evidence that addiction to video games is linked to worse memory, attention, as well as cognitive and academic skills in school children. This analysis constitutes a vital first step towards a better understanding of video games addiction in children, supporting its evidence as a plausible entity with detrimental association. By this, the study encourages parents of children addicted to video games to set particular gaming rules (such as limiting gaming time and avoiding exploration of new games), advocates schools to introduce children to other fun activities to do, and highlights the importance of consulting a therapist in extreme cases.

## Limitations

There are several limitations that should be noted. First of all, all scales were self-rated, which may only show high risk of addiction to video gaming rather than the diagnosis. Second, residual confounding bias is also possible, since there could be factors related to video gaming such as pre-existing attention problems, ADHD diagnosis that were not measured in this study. Moreover, parents might have over-or underestimated the answers to the questions, possibly predisposing us to information bias. A selection bias is also possible because of the enrollment of the students from three schools only and because of the convenient sample. The group of students chosen in this study were not representative of most of students in their age. Students’ parents filled up the video gaming addiction scale, which could lead to information bias since this scale is not applicable to them. To add on that, since this study is cross-sectional, the findings of this study cannot address the causal relationships among the primary constructs of interest. Finally, the scales used have not been validated in Lebanon. The use of a random sample is an important advantage to the study, thus, the results we obtained can be generalized to the whole population. The results cannot be generalized to the whole population since it only recruited students from private schools, as well as a high percentage of well-educated mothers. A prospective longitudinal study is needed to better follow up the cognitive function of children over time.

## Conclusion

In conclusion, the results suggest a correlation between addiction to video games and worse memory, attention, as well as cognitive and academic abilities among school children. Those findings indicate the need for more extensive research, and serve to highlight vital next steps needed in future papers, such as identifying predicting factors that could aid in early detection of video gaming addiction in children.

## Data Availability

There is no public access to all data generated or analyzed during this study to preserve the privacy of the identities of the individuals. The dataset that supports the conclusions is available to the corresponding author upon request.

## Abbreviations

DSM-5: Diagnostic and Statistical Manual of Mental Disorders
ICD-11: 11th Revision of International Classification of Diseases
ADHD: Attention deficit hyperactivity disorder
GASC: Game Addiction Scale for Children
CMQ: Children’s Memory Questionnaire
LEAF: Learning, Executive and Attention Functioning Scale
MANCOVA: Multivariate analysis of covariance
